# Coping strategies, stress, physical activity and sleep in patients with unexplained chest pain

**DOI:** 10.1186/1472-6955-5-7

**Published:** 2006-10-31

**Authors:** Margaretha Jerlock, Fannie Gaston-Johansson, Karin I Kjellgren, Catharina Welin

**Affiliations:** 1 Institute of Health and Care Sciences, The Sahlgrenska Academy, Göteborg University, Sweden; 2 Johns Hopkins University, School of Nursing, Baltimore, USA

## Abstract

**Background:**

The number of patients suffering from unexplained chest pain (UCP) is increasing. Intervention programmes are needed to reduce the chest pain and suffering experienced by these patients and effective preventive strategies are also required to reduce the incidence of these symptoms. The aim of this study was to describe general coping strategies in patients with UCP and examine the relationships between coping strategies, negative life events, sleep problems, physical activity, stress and chest pain intensity.

**Method:**

The sample consisted of 179 patients younger than 70 years of age, who were evaluated for chest pain at the emergency department daytime Monday through Friday and judged by a physician to have no organic cause for their chest pain. The study had a cross-sectional design.

**Results:**

Emotive coping was related to chest pain intensity (r = 0.17, *p *= 0.02). Women used emotive coping to a greater extent than did men (*p *= 0.05). In the multivariate analysis was shown that physical activity decreased emotive coping (OR 0.13, p < 0.0001) while sex, age, sleep, mental strain at work and negative life events increased emotive coping. Twenty-seven percent of the patients had sleep problems 8 to14 nights per month or more. Permanent stress at work during the last year was reported by 18% of the patients and stress at home by 7%. Thirty-five percent of the patients were worried often or almost all the time about being rushed at work and 23% were worried about being unable to keep up with their workload. Concerning total life events, 20% reported that a close relative had had a serious illness and 27% had reasons to be worried about a close relative.

**Conclusion:**

Our results indicated that patients with more intense UCP more often apply emotive coping in dealing with their pain. Given that emotive coping was also found to be related to disturbed sleep, negative life events, mental strain at work and physical activity, it may be of value to help these patients to both verbalise their emotions and to become cognizant of the influence of such factors on their pain experience.

## Background

Chest pain is one of the most common symptoms prompting individuals to seek acute care; however, more than half of the patients will not be judged to have chest pain of a cardiac origin [1,2]. In Sweden, this patient group has increased dramatically during the last fifteen years [3].

The unexplained chest pain (UCP) is often experienced for more than three months as was shown in two qualitative studies [4,5] that included 20 subject each. The International Association for the Study of Pain (IASP) [6] makes a distinction between acute and chronic pain. The definition of chronic pain is based on duration of pain for three months or more. According to the IASP [6] definition of pain, UCP constitutes chronic pain.

Unexplained chest pain is complex and gives rise to feelings of fear and anxiety, uncertainty, stress and loss of strength. The chest pain often persists, resulting in long term physical limitations and impairment of daily activities [4,5]. Patients with constant pain, despite reassurance that they do not have heart disease, are known to have poor overall outcome in terms of symptom distress, disability, and continuing concern about heart disease. Karlson et al. [2] investigated symptoms and well being in an unselected patient group (n = 1090) where acute myocardial infarction (AMI) was not confirmed, one year after discharge form hospital. They found that patients without AMI had more sleep problem, fatigue, chest pain, limitations in daily life than patients with confirmed AMI. Asbury et al. [7] compared women diagnosed with syndrome X (n = 100) and coronary heart disease (n = 100) and healthy controls (n = 100) concerning anxiety, depression, social support and life events. The results showed that patients with syndrome X had more anxiety, were more depressed than controls and felt that their health interfered more with everyday life than women in the other two groups. However, there were no differences concerning life events. Lau et al[8] compared the occurrence of daily hassles and major life events in five groups of subjects (28 patients with atypical chest pain without gastro-oesophageal reflux, 20 with atypical chest pain and oesophageal reflux, 33 with dyspepsia, nine patients with obstructive airway disease, peptic ulcers and gallstone, and 33 healthy controls) and found that patients with atypical chest pain without underlying motility/reflux abnormalities had higher scores of negative life events and daily hassles than the other four groups.

Bradley et al [9] compared psychosocial factors in five groups of patients (24 patients with chronic UCP, 15 with irritable bowel syndrome (IBS), 32 with gastro-oesophageal reflux (GERD), 12 with coronary artery disease (CAD), and 21 healthy controls) and the results showed that patients with chronic UCP had a greater use of maladaptive strategies than patients with CAD and IBS. Cheng et al. [10] found in their study that the patients with UCP (n = 70) used problem-focused strategies, but had an inflexible coping style compared to patients with rheumatism (n = 70) and healthy individuals (n = 70). Coping strategies in patients with chronic pain have been investigated by Hallberg et al. [11], who found that patients with fibromyalgia seem to interpret stressful situations as more threatening and to engage in more catastrophic thinking than do patients with work-related muscular pain. Woby et al. [12] investigated 90 patients with chronic back pain and found that patients with passive coping strategies, such as praying and hoping, evaluated the chest pain intensity to a higher level.

Sleep problems are a frequent complaint in patients with chronic pain. Sleep is important for health and is related to physical and emotional well-being. Hack and Mullington [13] investigated 40 individuals in a random controlled in-laboratory study. The results from this study showed that chronic insufficient sleep may affect health by bodily discomfort, fatigue, anger-aggression, body pain, stomach pain and backache. Asplund and Åhberg investigated women in a survey and showed a relationship between disturbed sleep and the occurrence of cardiac symptoms [14].

Since psychosocial factors are related to chest pain there is a need for treatments that complement customary medical care for UCP patients [15-18]. To date, systematic descriptions of psychosocial factors in UCP patients are lacking in the literature. Such investigations may provide important information for developing treatment strategies to decrease pain and improve quality of life in these patients.

The aim of this study was to describe general coping strategies in patients with UCP and examine the relationships between coping strategies, negative life events, sleep problems, physical activity, stress and chest pain intensity.

## Method

Present study had a cross-sectional design and was carried out at Sahlgrenska University Hospital in Göteborg, Sweden from December 2002 to September 2003, with a five-week break in June–July. The study was approved by The Ethics Committee of Göteborg University, Sweden (Study code 169-02) and carried out in accordance with the Declaration of Helsinki.

### Study Sample

Patients younger than 70 years of age who were evaluated for chest pain at the emergency department (ED) daytime Monday through Friday and judged by a physician to have no organic cause for the chest pain were included (i.e. gastro-oesophageal, cardiac or lung diseases were excluded). In total, 285 patients were eligible during the study period. Patients were excluded who 1) were identified for transfer to intensive care or were too ill to take part in the pain assessment or 2) had language difficulties. In total, 179 patients (73.4%) participated in the study (table 1).

**Table 1:** Study population. Participants and non-participants.

	**Men**		**Women**		**Total**	
	n	(%)	n	(%)	n	(%)
Eligible	166		119		285	
Excluded						
Too ill	2	(1.2)	2	(1.7)	2	(1.4)
Language difficulties	25	(15.1)	12	(10.1)	37	(13.0)
Included	139	(83.7)	105	(88.2)	244	(85.6)
Refused to participate	10	(7.2)	8	(7.6)	18	(7.4)
Administrative reasons^a^	28	(20.1)	19	(18.1	47	(19.3)
Participants	101	(72.7)	78	(74.3)	179	(73.4)

^a ^Patients missing due to an overcrowded ED.

### Procedure

All eligible patients were met by a nurse and assessed according to routines at the ED. Blood pressure, pulse, and a standard 12-lead electrocardiogram were taken and subsequently one of the two investigators in the research project asked the patients to participate in the study. The patients received both written and verbal information about all steps in the study from the investigator. After written informed consent was obtained, a pain assessment was performed on each patient by the investigator. The patients also filled in a questionnaire concerning psychosocial factors before discharge from ED.

### Pain assessment

The Pain-O-Meter (POM) is based on the gate control theory [19] and is designed to assess the intensity, duration, location and quality of pain. In this study, pain intensity was assessed by using the100-mm vertical visual analogue scale (VAS) on the POM, with anchors of no pain at the bottom and excruciating pain at the top. The reliability and validity of the POM-VAS are well documented and a test-retest correlation of r = 0.88 (*p *< .001) has been reported for intensity ratings of the POM-VAS [20].

### Coping measurement

According to Lazarus and co-workers [21], coping is a multidimensional process of cognitive and behavioural efforts to master, tolerate or reduce external and internal demands or conflicts created by stressful situations. They describe two main strategies, problem focused and emotion-focused strategies. Problem-focused coping is used when the problem is experienced as changeable and controllable. Emotion-focused coping is used when the situation is uncontrollable and too difficult to handle and solve. The goal of this strategy is to relieve the emotional impact of stress and make the situation more tolerable [21].

Jalowiec Coping Scale (JCS) [22] was used to assess general coping strategies in order to find out if coping strategies were related to the chest pain intensity. This 40-item questionnaire is based on Lazarus model of coping and was developed to cover and assess a wide range of coping behaviours. Jalowiec included a third concept, palliative coping which is a way of modulating tension and making the situation more tolerable or keeping it under control without directly taking care of the problem. Ten of the items in the subscale were previously classified as affective coping strategies and four as problem-oriented. Each item assesses how often a strategy is used to handle stress. Items are rated on a 4-point response scale ranging from never (1), sometimes (2), often (3) to almost always (4). The instrument assesses the variety of cognitive and behavioural strategies that subjects use to reduce stress. Factor analysis of the JCS has yielded a three-factor structure solution with three independent coping dimensions labelled confrontive coping (COS; 13 items, score range 13–52), emotive coping (EOS; 9 items, score range 9–36) and palliative coping (POS; 14 items, score range 14–56). Confrontive coping strategies focus on constructive handling of the stressful situation and facing up to the problem. Emotive coping strategies express emotions evoked by the situation. Jalowiec has comprehensively evaluated the construct validity of the JCS and has reported adequate internal consistency for the three factors (Cronbach's alpha 0.70–0.85) [22]. The instrument has been translated from English into Swedish and used in a Swedish patient population [23].

### Jenkins sleep scale

Sleep problems were assessed using Jenkins Sleep Scale [24]. This questionnaire comprises 4 items rated on a 6-step Likert scale, where 0 corresponds to no problems with sleep and 5 indicates sleep problems (scale score 0–20). The internal consistency of the sleep scale has been tested in a sample of British civil servants (Cronbach's alpha = 0.77) [25] and in the present study the alpha value was 0.80. The four items ask how often during the last month the subject has experienced the following: trouble falling asleep, trouble staying asleep, waking up several times per night, and waking up feeling tired and worn out after usual amount of sleep. The response alternatives are: not at all (0), 1–3 days (1), 4–7 days (2), 8–14 days (3), 15–21 days (4), 22–31 days (5). Patients are also asked to indicate their average number of hours sleep per night.

### Stress at home and at work

Psychological stress was assessed with two questions related to stress at work and home. Stress was defined as feeling irritable, filled with anxiety or having sleep problems. The participants were asked to report how often they had felt stress on a 6-step scale: never experienced stress (0), experienced some period of stress (1), some period of stress in the last five years (2), several periods of stress in the last five years (3), permanent stress in the last year (4) and permanent stress in the last five years (5). A summary score is obtained by averaging the ratings from the two questions (scale score = 0–5). These two questions are based on one single question combining stress at work and home used in numerous studies in Göteborg, Sweden, e.g. in a case-control study in 52 countries on psychosocial risk factors and acute myocardial infarction [26].

Mental strain at work was assessed with two questions: "How often do you worry about being rushed at work?" and "How often do you worry about keeping up with your workload" Ratings are made on 5-step response scale: never (1), seldom (2), sometimes (3), very often (4) and almost all the time (5). A summary score is obtained by summing the ratings of the two questions (range = 2–10). These two questions have been used in a case-control study in patients with acute myocardial infarction [27] and Cronbach's alpha for the referents (n = 412) was 0.91 for the scale. For the UCP patients in the present study, the Cronbach's alpha coefficient was 0.76.

### Physical activity

Physical activity in leisure time was assessed on a 4-step scale: sedentary (1) moderate physical (2) regular physical activity (3) and hard training or competitive sports (4) [28].

### Life events

Negative life events were assessed in relation to the following 10 events: serious illness in a close relative; death of a close relative; worry about a close relative; divorce; forced to move to a new home: forced to change jobs; unemployment; insecurity at work; serious financial worries; an event leading to legal consequences. The response alternatives were: no (0); yes, earlier (1); yes, during the last year (2); yes, both during the last year and earlier (3) (scale score 0–30). The instrument has been used in patients with acute myocardial infarction [27].

### Statistical analysis

All statistical analyses were performed in SAS (Statistical Analysis System) version 8.2 (SAS, Cary, NC, USA). Standard descriptive statistics were used. Spearman's correlation coefficient was used to evaluate relationships between selected psychosocial factors and chest pain intensity. P-value < 0.05 was accepted as statistically significant. To test the internal consistency of the scales, Crohnbach's alpha was computed. Odds ratios were calculated by a logistic regression model in order to determine the relationship between coping strategies and demographics, sleep problems, life events, physical activity and mental strain at work.

## Results

Of the 179 participating patients, 56.4% were men. The mean age for men was 44.2 (SD 13.0; range 19–67) and 46.6 (SD 13.3; range 17–69) for women. There was no significant age difference between men and women. Thirty-four percent of the patients were immigrants; the average length of residence in Sweden was 23 years (SD ± 13) for men and 25 years (SD ± 14) for women. Patient characteristics are presented in table 2. Table 3 shows mean, standard deviation and differences in men and women of the variables in the study.

**Table 2:** Demographic variables and traditional risk factors for ischemic heart disease in patients with UCP.

	**Men **n = 101		**Women **n = 78		**Total **n = 179	
Variables	%	(n)	%	(n)	%	(n)
Single	26	(26)	31	(29)	27	(49)
University education	23	(23)	22	(17)	22	(40)
Working full time or part time	73	(74)	63	(49)	69	(123)
Sick leave during the last year	25	(25)	29	(22)	27	(47)
Immigrant	35	(35)	33	(25)	34	(60)
Present smoker	17	(17)	35	(27)	25	(44)
Sedentary in leisure time	27	(27)	21	(16)	24	(43)
Body mass index ≥ 25	70	(69)	45	(34)	59	(103)
Diabetes	5	(5)	10	(8)	7	(13)
Hypertension	20	(20)	32	(25)	26	(45)
Myocardial infarction in one/both parents	31	(30)	34	(26)	32	(56)
Intake of analgesics, once a week or more	11	(10)	11	(8)	11	(18)

**Table 3:** Mean (SD) and differences in men and women with UCP.

	**Men **(n = 101)		**Women **(n = 78)		
	Mean	(SD)	Mean	(SD)	*p*-value
Age	44.2	(13.0)	46.6	(13.3)	0.22
Physical activity (1–4)	2.0	(0.8)	1.9	(0.5)	0.19
BMI	26.9	(3.9)	26.3	(6.1)	0.39
Chest pain intensity (0–100 mm)^a^	61	(23)	65	(25)	0.27
Confrontive coping (0–52)	31.1	(7.4)	30.8	(5.6)	0.81
Emotive coping (0–36)	17.0	(4.5)	18.4	(3.6)	0.03
Palliative coping (0–56)	27.2	(5.0)	28.1	(4,9)	0.21
Negative life events (0–30)	5.8	(4.2)	6.3	(4.5)	0.45
Stress at home (0–5)	1.8	(1.5)	1.9	(1.6)	0.68
Sleep problems (0–20)	7.9	(5.2)	8.8	(5.0)	0.27
Mental strain at work (2–10)	6.1	(2.3)	6.0	(2.1)	0.82

^a^During the last 24 hours

### Prevalence of pain

The patients were asked to indicate the intensity of their worst pain during the last 24 hours. The median value for chest pain intensity was 60 (range 5–100) in men and 70 (range 1–100) in women.

### Coping strategies

There were no significant gender differences in the use of confrontive (COS) or palliative (POS) coping strategies, but women used emotive (ECS) coping strategies to a greater extent than did men (*p *= 0.05). Table 4 presents the most frequently endorsed items (rated sometimes, often, or almost always) in each of the coping strategies used by men versus women.

**Table 4:** Coping strategies in patients with UCP. The most frequency items, and items with gender differences.

	**Men **(n = 101)		**Women **(n = 78)		
	%	(n)	%	(n)	*p*-value
CONFRONTIVE COPING (13 items^a^)					
*Most frequent*^b^					
Think through different ways to manage the situation	92	(91)	100	(72)	0.01
Try to keep the situation under control	95	(92)	93	(65)	0.59
Actively try to change the situation	88	(85)	96	(66)	0.11
Try to look at the problems objectively and see all sides	87	(84)	92	(65)	0.31
Try out different ways of solving the problems	84	(82)	94	(65)	0.05
Try to find out more about the situation	79	(77)	91	(63)	0.04
*Significant gender differences*^b^					
Seek support from family or friends	66	(65)	86	(63)	0.002
					
EMOTIVE COPING (9 items^a^)					
*Most frequent*^b^					
Worry	95	(94)	100	(75)	0.05
Get nervous	77	(76)	96	(70)	0.001
Take off by myself, want to be alone	72	(71)	84	(62)	0.06
*Significant gender differences*^b^					
Get angry	50	(50)	72	(52)	0.004
Eat, smoke, chew gum	49	(49)	68	(50)	0.02
					
PALLIATIVE COPING (14 items^a^)					
*Most frequent*^b^					
Hope for improvement	93	(92)	96	(71)	0.40
Accept the situation as it is	90	(88)	90	(61)	0.98
Try to put the problems out of my mind	90	(89)	94	(69)	0.27
Laugh it off and think things could have been worse	85	(83)	90	(65)	0.28
Settle for the next best thing to what I really want	74	(72)	80	(55)	0.41
*Significant gender differences*^b^					
Resign because things look hopeless	50	(49)	71	(49)	0.01
Pray and trust in God	34	(34)	44	(32)	0.20
Resign because it is fate	34	(33)	47	(32)	0.08
					
OTHER (4 items^a^)					
Cry^b^	30	(30)	81	(60)	<0.0001
Drink alcohol^b^	44	(43)	38	(28)	0.43
Meditate, use yoga or other relaxation^b^	20	(19)	40	(28)	0.004
Take medication to reduce tension^b^	18	(18)	19	(14)	0.86

^a^Response alternatives: Never, Sometimes, Often, Almost always

^b^Sometimes, Often or almost always

### Life events

The most common life events in both men and women were those related to close relatives. Concerning total life events (last year and earlier), 20% (n = 35) of the patients reported that a close relative had had a serious illness, 27% (n = 49) had had reasons to be worried about a close relative and 11% (n = 19) reported that a close relative had died.

### Stress at work/home

Permanent stress at work during the last year was reported by 18% of the patients (n = 31) and during the last five years by 11% (n = 20). Permanent stress at home during the last year was reported by 7% (n = 13) and during the last five years by 8% (n = 14).

Concerning mental strain at work, 35% (n = 63) of the patients were worried often or almost all the time about being rushed at work and 23% (n = 41) were worried about being unable to keep up with their workload.

### Sleep

Twenty-seven percent (n = 47) of the patients had sleep problems more than 14 nights per month (Jenkins sleep scale score ≥ 12; range 0–20). Twelve percent (n = 22) of the patients slept 5 hours or less, 26% (n = 47) slept 6 hours and 61% (n = 109) slept 7 hours or more.

### Correlations between included variables

Table 5 shows correlations between included variables. Emotive coping was correlated to all psychosocial factors. Negative life events were correlated to stress at home, sleep problems, palliative coping strategies and immigration. Sleep problems were correlated to negative life events, mental strain at work, stress at home and emotive coping.

**Table 5:** Correlations between included variables among patients with unexplained chest pain (n = 179).

		2.	3.	4.	5.	6.	7.	8.	9.	10.	11.	12.	13.	14.	15.	16.
1.	Single	-0.04	-0.07	-0.04	0.03	-0.05	-0.07	-0.02	-0.04	0.16*	-0.03	-0.02	-0.13	0.11	-0.02	0.19
2.	University education		-0.13	-0.62	0.01	-0.14	0.23**	-0.12	-0.05	0.25**	-0.17*	-0.12	-0.11	-0.00	-0.11	0.11
3.	Working full time/part time			0.00	0.20**	-0.88	-0.03	0.09	0.11	0.08	0.14	0.26**	0.15*	0.13	0.04	0.17*
4.	Sick leave the last year				0.03	0.12	0.09	0.09	0.08	-0.06	0.04	0.06	0.13	0.15*	0.09	-0.01
5.	Immigrant					-0.03	0.16*	0.06	0.11	-0.02	0.16*	0.35**	-0.02	0.24**	0.12	0.21**
6.	Present smoker						-0.31**	-0.16*	0.23**	-0.07	0.20*	0.08	0.16*	0.05	0.12	-0.02
7.	Physical activity							-0.11	-0.06	0.19*	-0.37**	0.04	-0.08	0.03	-0.23**	-0.03
8.	BMI								-0.02	0.02	0.17*	0.02	0.06	0.06	0.18*	0.13
9.	Chest pain intensity^a^									-0.05	0.17*	0.07	0.13	0.11	0.02	-0.02
10.	Confrontive coping										-0.12	0.11	-0.8	0.18*	-0.35	0.02
11.	Emotive coping											0.43**	0.36**	0.30**	0.35**	0.30**
12.	Palliative coping												0.14	0.17*	0.16	0.14
13.	Sleep problems													0.26**	0.18*	0.22**
14.	Negative life events														0.15	0.26**
15.	Mental strain at work															0.28**
16.	Stress at home															

^a^During the last 24 hours

* <0.05 ** <0.01

### Relationships between coping strategies, psychosocial factors and chest pain intensity

Spearman's correlation coefficient was used to evaluate univariate relationships between worst chest pain intensity in the last 24 hours, demographics and psychosocial factors. The only significant relationship was found between increased chest pain and emotive coping with a correlation of r = 0.17 (*p *= 0.02).

Odds ratios were calculated by a logistic regression model. Univariate and multivariate analysis were conducted to investigate how emotive coping was related to demographics and psychosocial variables in the study. Emotive coping was negatively related to physical activity (OR 0.13, *p *< 0.0001) and positively to sex, age, sleep, mental strain at work and negative life events (Table 6).

**Table 6:** Univariate and multivariate relationships between emotive coping strategies and psychosocial variables in patients with UCP (n = 179).

	**Emotive coping**					
	Univariate			Multivariate^a^		
Variables	OR	(95% CI)	*p*-value	OR	(95% CI)	*p*-value
Sex (female)	4.07	(1.12–14.79)	0.03	3.39	(1.01–11.40)	0.005
Age	0.94	(0.90–0.99)	0.02	0.93	(0.88–0.97)	0.004
Single (0–1)	1.17	(0.28–4.93)	0.83			
University education (0–1)	0.34	(0.07–1.63)	0.17			
Immigrants (0–1)	0.31	(0.08–1.19)	0.08			
Physical activity (1–4)	0.08	(0.04–0.21)	<0.0001	0.13	(0.06–0.32)	<0.0001
Sleep problems (0–20)	1.35	(1.20–1.51)	<0.0001	1.25	(1.11–1.42)	0.0002
Mental strain at work (2–10)	2.00	(1.48–2.70)	<0.0001	1.44	(1.08–1.91)	0.005
Stress at home (0–5)	2.17	(1.43–3.29)	0.0003	1.15	(0.74–1.77)	0.89
Life events (0–30)	1.29	(1.12–1.49)	0.0006	1.21	(1.04–1.41)	0.01

^a^Only significant (p < 0.05) variables in the unvariate analysis are included.

## Discussion

Our patients used confrontive strategies in stress situations, but the results suggest that they had difficulties to handle stress-related emotions, which affect the chest pain intensity. The results showed that there was a relationship between emotive coping and chest pain intensity. Our results also showed that negative life events and mental strain at work and sleep problems were positively related to emotive coping while physical activity was negative related to emotive coping. It may therefore be of great value to focus on emotional responses to stress in UCP patients and to help these patients to verbalise their emotions and to become cognizant of the psychosocial influences on the pain experience.

Similar results to our own were reported by Raak and Wahren [29] in women with headache e.g. patients who had experienced headache during the previous three months more frequently used emotive coping strategies than those with no headache during the same period.

Our multivariate analysis showed that physical activity independently predicted the use of emotive coping, where more physical activity was associated with less use of emotive coping. More than half of the patients had BMI ≥ 25 and improved physical activity could probably help these patients in losing weight. We know from previous research that physical activity reduces risk for ischemic heart disease (IHD) and Wilhelmsen *et al*. [30] have shown that patients with unspecific chest pain are at increased risk of dying from coronary heart disease. Improving physical activity in this patient group may decrease emotive coping and by that the chest pain intensity as well as risk factors for IHD.

Tyni-Lenne [31] investigated the effects of physical training in 24 women with syndrome X and found that the women benefit from exercise capacity and quality of life. In a previous qualitative study by Jerlock et al. [5], patients with UCP stated that they did not exercise because they were uncertain about how much they could exert themselves with their undiagnosed chest pain.

Sleep was also found to be related to emotive coping. In a study by Welin et al[32], 7% of men and 20% of women referents scored ≥ 12 on the Jenkins Sleep Scale, which is much less than among our UCP patients (27%). In a survey with 3669 randomly selected women, Asplund and Åberg [33] showed that all types of sleep disturbances, such as difficulty in falling asleep, early awakening and nightmares, increase the risk of cardiac symptoms as spasmodic chest pain and irregular heart beats. Less than 5 h sleep was associated with twofold increase in spasmodic chest pain.

Sedentary in leisure time and sleep problems were related to emotive coping and may therefore be important factors to pay attention to in efforts to alleviate chest pain. Studies have shown that regular exercise, such as walking, improves sleep [34,35]. In the above mentioned study by Jerlock et al[5], patients viewed walking as a safe form of exercise and walked long distances without problems. In intervention programmes, it is important that the patients be taught about the chest pain itself and how to handle situations to ensure that the patients feel secure when exercising.

Many UCP patients suffer from their symptoms for a long time [4,5]. In discussing the relationship between sleep and chronic pain, Roehrs and Roth [36] and Smith and Haythornthwaite [37] have pointed out that this relation is bidirectional and therefore treatment has to focus on both pain and sleep problems. Thirty-eight percent of our UCP patients reported 6 hours sleep or less per night. Roehrs *et al*. [38] conducted a study with a two repeated-design in a sleep laboratory. Thirteen pain-free individuals participated and the result showed that even modest shortened sleep produces hyperalgesia the following day.

The number of patients suffering from UCP is increasing and, correspondingly, the costs for caring for these patients are escalating, since many of them are admitted to the emergency ward overnight to rule out cardiac causes to the pain. It is therefore important to develop not only effective treatment strategies to reduce the suffering experienced by these patients, but also preventive strategies to reduce the costs for society.

The main limitation of the present study is its cross-sectional design which only assesses the use of coping strategies at only one point in time. The data were also collected at a single hospital at daytime, which means that our results are not generalisable to patients who seek care at night. In order to further explore the influence of psychosocial factors on UCP, we plan to conduct a case-control study comparing patients with UCP with a referent group to determine if patients with UCP differ from a healthy population regarding psychosocial factors.

### Implications for clinical practice

There is a lack of intervention studies concerning UCP patients which can be explained by a gap in the knowledge about this patient group, but also by difficulties to find robust methods to reduce biasis and to enhance the generalizability of the findings. Esler and Bock [18] describe the current state of treatment for non-cardiac chest pain (NCCP) and recommend a brief intervention which is able to reduce the frequency of NCCP. They recommend a bio-psychosocial model based on the knowledge that almost all illnesses are influenced by biological, psychological and social factors and propose that better outcomes are achieved when therapeutic interventions take into account the relationships between these factors. They suggest that treatment must focus on educating the patients to manage their chest pain symptoms, cardiac risk factors and stress.

Based on the results from this study, an intervention programme with a bio-psychosocial model could be appropriate in order to enhancing the patients coping ability since the emotive coping style seems to increase the chest pain intensity. A programme, containing education about chest pain, improved awareness about own coping strategies and reactions on stress situations, and the importance of physical activity could be valuable to decrease pain and improve the quality of life for these patients.

Intervention programmes should be designed to be applicable to the entire UCP patient group, but implemented in such a way that the needs of the individual patient are met. This means that the nurse must listen to how the patient describes and manages everyday life and promote communication conducive to learning [39].

## Conclusion

Our results indicate that the intensity of unexplained chest pain is related to emotive coping, where patients with more intense UCP more often apply emotive coping in dealing with their pain. Given that emotive coping was also found to be related to negative life events, mental strain at work, disturbed sleep and physical activity, it may be of value to help these patients to both verbalise their emotions and to become cognizant of the influence of such factors on their pain experience.

## Competing interests

The author(s) declare that they have no competing interests.

## Authors' contributions

MJ contributed in all stages of the research: planning, data collection and analyses, and in writing the bulk of the paper. FG-J initiated the research project. KK contributed in the analyses and writing. CW contributed in all stages of the research project. All authors have read and approved the final manuscript.

## Pre-publication history

The pre-publication history for this paper can be accessed here:


